# Supplementary material for: The Effect of Ground Type on the Jump Performance of Adults of the Locust Locusta migratoria manilensis: A Preliminary Study
Source: Insects. 2020 Apr 23;11(4):259. doi: 10.3390/insects11040259 (PMC7240473; doi:10.3390/insects11040259)
Supplement: Supplementary file 1 [file insects-11-00259-s001.zip › supplementary materials.docx]

Supplementary materials

The Effect of Ground Type on the Jump Performance of Adults of the Locust *Locusta migratoria manilensis*: A Preliminary Study

Chao Wan ^1,2,^*, Rentian Cao ^1^ and Zhixiu Hao ^1,^*

^1^ State Key Laboratory of Tribology, Tsinghua University, Beijing 100084, China;

^2^ Department of Mechanics, School of Aerospace Engineering, Beijing Institute of Technology, Beijing 100081, China

***** Correspondence: [chaowan@bit.edu.cn](mailto:chaowan@bit.edu.cn) (Chao Wan); [haozx@tsinghua.edu.cn](mailto:haozx@tsinghua.edu.cn) (Zhixiu Hao)

**Table S1. Experimental data of the kinematics for the locusts jumping on sand.**

| Sample No. | Sex | Body mass (g) | $\theta$ (degree) | $\beta_{\text{e}}$ (degree) | $\gamma$(degree) | $\beta_{\text{t}}$ (degree) | $S_{\text{d}}$ (cm) | $V_{\text{t}}$ (m/s) | $E_{\text{m}}$ (mJ/g) |
| --- | --- | --- | --- | --- | --- | --- | --- | --- | --- |
| 1 | Female | 2.54 | 17.3 | 65.8 | 55.0 | 49.8 | 45.0 | 2.12 | 2.24 |
| 2 | Female | 2.48 | 15.0 | 59.0 | 47.0 | 40.7 | 52.0 | 2.27 | 2.58 |
| 3 | Female | 2.33 | 24.3 | 60.8 | 47.2 | 38.4 | 42.8 | 2.08 | 2.15 |
| 4 | Female | 1.59 | 30.0 | 47.1 | 41.0 | 31.8 | 49.6 | 2.33 | 2.71 |
| 5 | Male | 1.45 | 26.6 | 49.4 | 40.2 | 23.5 | 68.9 | 3.04 | 4.62 |
| 6 | Male | 1.41 | 12.5 | 49.9 | 35.1 | 30.0 | 32.0 | 1.90 | 1.81 |
| 7 | Male | 1.43 | 21.6 | 57.8 | 45.3 | 36.7 | 43.0 | 2.10 | 2.20 |
| 8 | Male | 1.32 | 15.0 | 56.6 | 29.9 | 24.5 | 48.2 | 2.50 | 3.13 |
| 9 | Male | 1.42 | 20.8 | 68.1 | 56.1 | 48.8 | 57.4 | 2.38 | 2.84 |

**Table S2. Experimental data of the kinematics for the locusts jumping on soil.**

| Sample No. | Sex | Body mass (g) | $\theta$ (degree) | $\beta_{\text{e}}$ (degree) | $\gamma$(degree) | $\beta_{\text{t}}$ (degree) | $S_{\text{d}}$ (cm) | $V_{\text{t}}$ (m/s) | $E_{\text{m}}$ (mJ/g) |
| --- | --- | --- | --- | --- | --- | --- | --- | --- | --- |
| 1 | Female | 2.54 | 25.5 | 48.7 | 48.5 | 43.2 | 50.2 | 2.22 | 2.46 |
| 2 | Female | 2.48 | 11.3 | 50.6 | 51.6 | 40.7 | 63.7 | 2.51 | 3.16 |
| 3 | Female | 2.33 | 17.3 | 52.0 | 50.1 | 42.4 | 61.3 | 2.46 | 3.02 |
| 4 | Female | 1.59 | 15.2 | 51.8 | 42.8 | 37.3 | 48.2 | 2.21 | 2.45 |
| 5 | Male | 1.45 | 21.5 | 56.3 | 63.4 | 53.1 | 48.1 | 2.22 | 2.46 |
| 6 | Male | 1.41 | 19.9 | 35.7 | 46.1 | 35.0 | 43.5 | 2.13 | 2.27 |
| 7 | Male | 1.43 | 22.8 | 45.9 | 52.3 | 40.4 | 50.8 | 2.24 | 2.52 |
| 8 | Male | 1.32 | 19.7 | 65.3 | 59.0 | 52.9 | 56.7 | 2.40 | 2.89 |
| 9 | Male | 1.42 | 18.6 | 64.7 | 46.3 | 41.2 | 57.0 | 2.37 | 2.82 |

**Table S3. Experimental data of the kinematics for the locusts jumping on wood.**

| Sample No. | Sex | Body mass (g) | $\theta$ (degree) | $\beta_{\text{e}}$ (degree) | $\gamma$(degree) | $\beta_{\text{t}}$ (degree) | $S_{\text{d}}$ (cm) | $V_{\text{t}}$ (m/s) | $E_{\text{m}}$ (mJ/g) |
| --- | --- | --- | --- | --- | --- | --- | --- | --- | --- |
| 1 | Female | 2.54 | 18.1 | 59.7 | 63.2 | 57.7 | 63.7 | 2.63 | 3.46 |
| 2 | Female | 2.48 | 23.2 | 70.4 | 73.1 | 67.8 | 50.6 | 2.66 | 3.54 |
| 3 | Female | 2.33 | 18.8 | 57.0 | 65.1 | 57.0 | 61.9 | 2.58 | 3.32 |
| 4 | Female | 1.59 | 19.6 | 53.1 | 57.1 | 52.0 | 55.0 | 2.36 | 2.78 |
| 5 | Male | 1.45 | 22.4 | 51.2 | 57.9 | 50.3 | 48.6 | 2.20 | 2.43 |
| 6 | Male | 1.41 | 18.5 | 62.2 | 66.1 | 60.2 | 64.2 | 2.70 | 3.64 |
| 7 | Male | 1.43 | 14.0 | 62.7 | 65.6 | 60.3 | 65.6 | 2.73 | 3.73 |
| 8 | Male | 1.32 | 14.3 | 88.0 | 89.0 | 87.0 | 3.32 | 2.50 | 3.13 |
| 9 | Male | 1.42 | 19.2 | 74.7 | 78.6 | 73.2 | 18.2 | 2.56 | 3.38 |

**Table S4. Experimental data of the reaction force for the locusts jumping on sand.**

| Sample No. | Sex | $M$ (g) | $F_{\text{a}}$ (mN/g) | $F_{\text{l}}$ (mN/g) | $F_{\text{n}}$ (mN/g) | $F_{\text{t}}$ (mN/g) |
| --- | --- | --- | --- | --- | --- | --- |
| 1 | Female | 2.54 | 35.0 | 5.80 | 47.4 | 59.2 |
| 2 | Female | 2.48 | 33.4 | 17.5 | 38.8 | 54.1 |
| 3 | Female | 2.33 | 39.2 | 14.8 | 39.8 | 57.8 |
| 4 | Female | 1.59 | 44.5 | 7.73 | 52.8 | 69.5 |
| 5 | Male | 1.45 | 35.7 | 29.1 | 42.5 | 62.7 |
| 6 | Male | 1.41 | 41.4 | 10.2 | 40.5 | 58.9 |
| 7 | Male | 1.43 | 41.2 | 20.2 | 31.2 | 55.5 |
| 8 | Male | 1.32 | 42.7 | 22.1 | 33.6 | 58.7 |
| 9 | Male | 1.42 | 26.7 | 11.4 | 27.8 | 40.2 |

**Table S5. Experimental data of the reaction force for the locusts jumping on soil.**

| Sample No. | Sex | $M$ (g) | $F_{\text{a}}$ (mN/g) | $F_{\text{l}}$ (mN/g) | $F_{\text{n}}$ (mN/g) | $F_{\text{t}}$ (mN/g) |
| --- | --- | --- | --- | --- | --- | --- |
| 1 | Female | 2.54 | 17.5 | 10.1 | 19.1 | 27.8 |
| 2 | Female | 2.48 | 47.2 | 41.7 | 45.6 | 77.8 |
| 3 | Female | 2.33 | 25.0 | 3.27 | 25.6 | 36.0 |
| 4 | Female | 1.59 | 46.1 | 16.9 | 44.9 | 66.6 |
| 5 | Male | 1.45 | 33.1 | 24.1 | 34.8 | 53.7 |
| 6 | Male | 1.41 | 25.2 | 19.8 | 30.7 | 44.4 |
| 7 | Male | 1.43 | 25.0 | 23.2 | 49.1 | 59.8 |
| 8 | Male | 1.32 | 35.6 | 25.0 | 36.4 | 56.8 |
| 9 | Male | 1.42 | 27.6 | 15.9 | 27.5 | 42.1 |

**Table S6. Experimental data of the reaction force for the locusts jumping on wood.**

| Sample No. | Sex | $M$ (g) | $F_{\text{a}}$ (mN/g) | $F_{\text{l}}$ (mN/g) | $F_{\text{n}}$ (mN/g) | $F_{\text{t}}$ (mN/g) |
| --- | --- | --- | --- | --- | --- | --- |
| 1 | Female | 2.54 | 22.9 | 16.0 | 26.5 | 38.5 |
| 2 | Female | 2.48 | 19.7 | 3.97 | 99.1 | 101 |
| 3 | Female | 2.33 | 25.7 | 13.6 | 20.9 | 35.8 |
| 4 | Female | 1.59 | 20.8 | 8.48 | 23.5 | 32.5 |
| 5 | Male | 1.45 | 20.0 | 26.1 | 26.9 | 42.4 |
| 6 | Male | 1.41 | 7.75 | 15.1 | 10.5 | 19.9 |
| 7 | Male | 1.43 | 25.2 | 15.6 | 46.3 | 55.0 |
| 8 | Male | 1.32 | 11.7 | 5.14 | 44.8 | 46.6 |
| 9 | Male | 1.42 | 25.4 | 47.5 | 86.7 | 102 |

**Video S1.** A typical video of the locust jumping on sand. The video was produced based on the high-speed camera images (1000 fps). The frame rate of the video is 30 fps, meaning that each second in the video represents to the real time of 0.03 s.

**Video S2.** A typical video of the locust jumping on soil. The video was produced based on the high-speed camera images (1000 fps). The frame rate of the video is 30 fps, meaning that each second in the video represents to the real time of 0.03 s.

**Video S3.** A typical video of the locust jumping on wood. The video was produced based on the high-speed camera images (1000 fps). The frame rate of the video is 30 fps, meaning that each second in the video represents to the real time of 0.03 s.
